# Contingency planning for a deliberate release of smallpox in Great Britain - the role of geographical scale and contact structure

**DOI:** 10.1186/1471-2334-10-25

**Published:** 2010-02-14

**Authors:** Thomas House, Ian Hall, Leon Danon, Matt J Keeling

**Affiliations:** 1 Warwick Mathematics Institute and Department of Biological Sciences, University of Warwick, Coventry, UK; 2 Microbial Risk Assessment, Health Protection Agency, Emergency Response Department, Porton Down, Wiltshire, UK

## Abstract

**Background:**

In the event of a release of a pathogen such as smallpox, which is human-to-human transmissible and has high associated mortality, a key question is how best to deploy containment and control strategies. Given the general uncertainty surrounding this issue, mathematical modelling has played an important role in informing the likely optimal response, in particular defining the conditions under which mass-vaccination would be appropriate. In this paper, we consider two key questions currently unanswered in the literature: firstly, what is the optimal spatial scale for intervention; and secondly, how sensitive are results to the modelling assumptions made about the pattern of human contacts?

**Methods:**

Here we develop a novel mathematical model for smallpox that incorporates both information on individual contact structure (which is important if the effects of contact tracing are to be captured accurately) and large-scale patterns of movement across a range of spatial scales in Great Britain.

**Results:**

Analysis of this model confirms previous work suggesting that a locally targeted 'ring' vaccination strategy is optimal, and that this conclusion is actually quite robust for different socio-demographic and epidemiological assumptions.

**Conclusions:**

Our method allows for intuitive understanding of the reasons why national mass vaccination is typically predicted to be suboptimal. As such, we present a general framework for fast calculation of expected outcomes during the attempted control of diverse emerging infections; this is particularly important given that parameters would need to be interactively estimated and modelled in any release scenario.

## Background

Stopping transmission of the smallpox (*variola*) virus amongst the human population was one of the greatest public health triumphs of the twentieth century; and yet since the events of 11 September 2001 the possibility of its reemergence has been under increased study [1]. Effective contingency planning for such a scenario requires knowledge of the optimal deployment and use of case isolation, contact tracing and vaccination. Since a series of controlled experiments to inform such decisions is not possible, we are forced to rely on mathematical modelling to improve the evidence base for emergency preparedness and response. Recent simulation models [2-5] typically conclude that, provided a given smallpox outbreak can be well controlled, the emphasis should be on case isolation, contact tracing and targeted vaccination rather than immediate country-level mass vaccination, due to the frequency of adverse effects from vaccine and the likely high efficacy of other measures. Targeting of vaccination can either be exclusively towards suspected cases found by contact tracing, or can also involve a policy of local 'ring vaccination' of the population in the vicinity known cases. Evidence exists that such ring vaccination may provide additional benefit over individually targeted measures [5]. However, two questions remain and form the main considerations of this paper: the exact conditions for which ring vaccination is optimal, and the scale at which such vaccination should take place--in particular we focus on the interaction between ring vaccination and contact tracing in a clustered network of contacts.

When considering optimal ring vaccination in any epidemiological context, the key issue is the spatial scale for intervention. This presents a technical challenge, since spatial models of disease transmission are typically much more complex than non-spatial models. Local vaccination is also not applied in isolation, but is deployed together with other control measures that must be captured by the model. One of the most widely used additional measures is contact tracing, which seeks to identify infected individuals before they become fully symptomatic by tracing the contacts (and therefore potential secondary cases) from each identified case. Theoretical work shows that failure to account for the underlying structure of the contact network when considering contact tracing can cause severe, qualitative errors in model results [6]. We therefore find that our model choice is constrained by the epidemiology, dynamics, and types of control measure that we wish to capture.

Three main model types have been used to investigate the spread and control of smallpox [7]. Mean-field models [[8], §8.3] have the advantage of being a relatively tractable set of differential equations, but suffer from the underlying assumption that transmission occurs at random within the entire population. Metapopulation models [5] are based on the observation that most infectious individuals create the majority of their secondary cases within their local environment; in these metapopulation models the population is therefore aggregated in local administration units with most transmission occurring within each unit and at random. Individual-based simulation models [2-4] are generally considered the most realistic and aim to capture the full contact structure between all individuals in the population, often assigning individuals to households, schools and workplaces where there is known to be a higher risk of transmission. Here we define a model that includes mixtures from all three of the above methods: it is based on differential equation and therefore is rapid to simulate allowing rigorous sensitivity analysis; it retains the metapopulation concept of local and longer-range transmission so can be used to consider regional (ring) vaccination; and it incorporates elements of local contact structure and can therefore reliably capture the effects of contact tracing.

Individual-based simulation can in principle incorporate any population structure and interventions necessary, and as such there will always be an important role in contingency planning for computationally intensive models that aim for maximum realism. Nevertheless, there are limitations to these approaches that motivate our methodology of developing a new, more parsimonious model. Most important for our purposes are the problems of parameterisation, numerical tractability and transparency. With such individual-based simulations there is always the temptation to include ever more complexity, however any model is necessarily a caricature of reality and so the quest for ever greater realism can never be fulfilled. While computers and algorithms continue to improve, increasing the number of individuals that can be directly simulated and decreasing the time to perform a simulation, considering the whole population of England, Wales and Scotland still involves significant computational resources. While baseline results can be obtained at these population sizes reasonably quickly, applications that require large number of model realisations such as comprehensive sensitivity analysis, real-time parameter fitting and determination of optimal strategies quickly become highly time-consuming. The development of models and methods to implement such applications remains necessary, however complementary approaches that are more abstract can significantly reduce the computational burden.

A model that incorporates synthetic data on individuals and their contacts almost by definition involves many more parameters than can be measured directly, and as such assumptions have to be made on the basis of available data. For example, if an explicit network is generated as in [9], then there is really a network parameter for every pair of individuals in the population modelled. Given the gap between available data on human contacts and the information contained in an explicit network, a complementary approach to individual-based simulation is to develop mathematical approaches that give epidemiological outputs based on statistical properties of contact networks. Such network properties can often be measured directly, and the uncertainty in them estimated systematically. One published methodology for this, applied to smallpox, is to use a branching-process model [10]. Such an approach does not, however, allow the consideration of clustering--the possibility that an individual's contacts also contact each other. Work not directly related to smallpox, based on pairwise models, has shown that clustering has a major impact on transmission and tracing dynamics [6,11,12]. This leads us to extend pairwise models to enable smallpox to be modelled whilst considering clustering. A thorough treatment of this methodology is available in [[8], Chapter 7] and [13].

## Methods

For reasons explained in the introduction above, we chose a modelling approach that uses mathematical techniques to reduce the computational burden. This means that the underlying model has simple underlying assumptions and relatively few parameters, which we outline below, but ultimately rests on specialist mathematical results, which we have included in Additional file 1. A full list of parameters, including references, is provided in Table 1. Our model is structured around the pairwise approach to capture the natural history of infection and control, together with the metapopulation concept of rare external transmission outside of local spatial units.

**Table 1:** Model parameters, together with baseline value and range if varied during analysis.

Parameter	Description	Value (Range)	Refs
*g*_*E*_	Rate of transition from latent to prodromal	1/12 days^-1^	[14]

*g*_*P*_	Rate of transition from prodromal to infectious	1/2.5 days^-1^	[15]

*g*_*I*_	Rate of transition from infectious to removed	1/8.6 days^-1^	[16]

*δ*	Case fatality risk	30%	[23,33]

*θ*	Probability of case isolation success	90%	[21]

*g*_*Q*_	Rate of transition from isolated to removed	1/20 days^-1^	[5]

ϵ	Probability of contact tracing success	80%	[5]

ϵ_1_	Vaccine efficacy when susceptible	97.5%	[14]

ϵ_2_	Vaccine efficacy when latent	30%	[23]

*g*_*O*_	Rate of transition from observed to removed or vaccinated	1/15 days^-1^	[5]

γ	Proportion of population contraindicated for mass vaccination	30%	[25]

*E*_0_	Number of index cases	10	see text

	Number of index cases for secondary outbreak	1	see text

*I*_trig_	Number of clinical cases prior to detection	4	see text

	Number of clinical cases prior to detection for secondary outbreak	1	see text

*δ*_*V*_	Vaccine fatality risk	10^-5^(0 -- 10^-5^)	[24]

*R*_0_	Basic reproductive ratio	5 (3 -- 7)	[14]

*R*_*P*_	Prodromal type reproductive ratio	0.5 (0.1 -- 1.5)	[15,17]

*κ*	Movement reductions when infectious	0.9 (0 -- 1)	[5]

*v*	Rate of mass vaccination	*N*/7 (see Figure 3(c))	see text

*N*	Population size of region	10^5^(see Figure 3(a))	[28,29]

*ξ*	Region outwardness	0.4 (see Figure 3(d))	[27,30]

*M*	Number of regions of same type as outbreak region	n/a (see Figure 3(a))	[28,29]

*M*_0_	Number of regions at relevant scale initially infected	1 (1 -- 10)	see text

*n*	Neighbourhood size (contacts per individual)	17 (5 -- 50)	[18,19]

*ϕ*	Clustering coefficient	0 (0 -- 0.5)	see text

Either references are given, or the parameter value is discussed in the main text. Values referenced to [5] are typically also based on expert opinion from discussions with the Department of Health. [34] provides an example of the considerations involved in estimating these values

### Smallpox natural history

**Figure 1:**
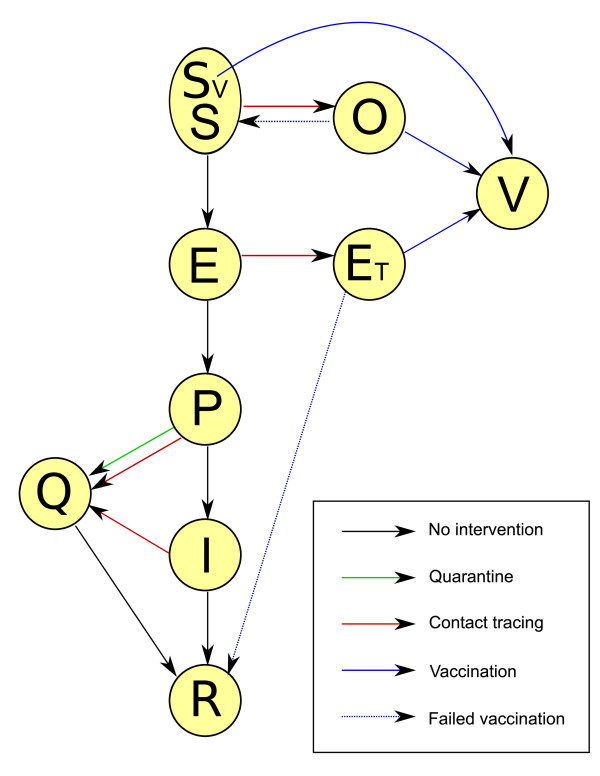
**Disease states of the model, and processes connecting them**. These are: *S *susceptible; ^*S*^*V *susceptible and not contraindicated for vaccination; *E *latent; *P *prodromal; *I *infectious; *R *removed; *Q *isolated; *O *susceptible under observation; ^*E*^*T *latent under observation; *V *vaccinated. The latent class is populated by transmission from infectious or prodromal cases to susceptibles.

The basic natural history of infection within our model is shown in Figure 1. At the centre of this are five disease states that smallpox cases pass through in the absence of any intervention. At first, individuals are susceptible (*S*), then following infection they are exposed (*E*) and asymptomatically incubate the infection. After the latent period, cases become prodromal (*P*) and exhibit non-specific influenza-like symptoms whilst being able to transmit infection, before quickly developing the characteristic rash of a fully infectious (*I*) case. Once recovered from infection, individuals play no further role in transmission and are removed (*R*) from the infection model, which can represent a range of outcomes. We assume that a proportion *δ *= 0.3 of cases die, and that others become fully recovered and immune.

The progression of disease following infection therefore involves three processes, which are assumed to happen at the following rates [14-16]:(1)

When we come to consider transmission, the most important parameter is the basic reproductive ratio, *R*_0_, which represents the expected number of secondary cases produced by a typical primary case early in the epidemic. Given that both prodromal and fully infectious individuals can transmit infection, we can split *R*_0 _= *R*_*P *_+ *R*_*I *_where *R*_*P *_is the expected number of secondary cases caused during the prodromal stage and *R*_*I *_the number during the fully infectious stage. Our default assumptions are that *R*_0 _= 5, *R*_*P *_= 0.1 × *R*_0_, *R*_*I *_= 0.9 × *R*_0 _[14,15,17].

### Contact network structure

The full network of human contacts capable of spreading infectious disease is undoubtedly complex, highly structured and dynamic. At the same time, there are many network statistics that are known to be epidemiologically important, however these are all based on the facts that transmission-relevant contacts are finite and non-random (captured by the mean number of contacts per individual, *n*) and that contacts are often shared by individuals (captured by the clustering coefficient *ϕ*). These two parameters are, therefore, an appropriate choice for our purposes and we vary them significantly during our analysis. We note that in many cases the heterogeneity in the number of contacts per individual can play an important role; but for the early stages of an outbreak considered here the effects of this heterogeneity can largely be subsumed into the parameterisation of *R*_0_.

The mean number of contacts, *n*, has been estimated and we take it to be 17 people as a default [18,19]. The clustering coefficient *ϕ*, which is equal to the mean proportion of shared contacts between two linked individuals, has yet to be measured in an accurate way, and so our baseline value is *ϕ *= 0 for comparison with existing unclustered models. Since we know that there are many internally well-connected groups and cliques within human populations, such as households, schools and workplaces, it is most likely that *ϕ *is far from 0 and so large values are considered when we generalise.

Introducing a notation in which [*S*] represents the number of susceptible individuals in the population, and [*SI*] the number of contacts between a susceptible and an infectious individual (and similarly for other disease states) we can now introduce rates for the transmission of infection across the contact network, *τ_P _*and *τ_I_*, which are defined by(2)

So prodromal individuals infect susceptible individuals that they are connected to at a rate *τ_P _*and infectious individuals infect susceptible individuals that they are connected to at a rate *τ_I _*.

If we were not considering a model with an explicit contact network, then the relationship between underlying transmission rates and observables can be dealt with in a rigorous way, such as in [20]. On a network, however, the relationship between transmission rates and the reproduction numbers *R*_0_, *R*_*P*_, *R*_*I *_discussed above is subtle, and the technical details of our approach are given in Additional file 1. The important conclusion from this technical development is that there are really two types of reproduction number that one should consider. The first, which we write *r*_0 _(composed of *r*_*I *_and *r*_*P*_), relates to population-level measurements of incidence and prevalence and their early growth rate, while the second, which we write ℛ_0 _(and is composed of ℛ_*I *_and ℛ_*P*_) relates to individual-level measurements made during contact tracing and is more true to the verbal definition. We consider both types of reproduction number in our analysis.

### Control strategies

We now consider methods and parameters for the various interventions that are typically considered in smallpox contingency planning.

#### Case isolation

The first step in outbreak containment is to isolate symptomatic cases as soon as possible, to stop transmission of disease. We model this by taking a proportion *θ *= 0.9 of the prodromal cases into a new quarantined class, *Q*, rather than the infectious *I *class at the end of the prodromal period [21]. Isolated cases are released into the recovered *R *class with rate constant *g*_*Q*_= (1/20) days^-1 ^when they are assumed to pose no further risk of transmission [5]. This measure on its own therefore reduces the number of secondary cases produced to *R*_*P *_+ 0.1 × *R*_*I *_= 1 under our baseline assumptions, which is consistent with existing work and expert opinion [22].

#### Contact tracing

Contact tracing of known (and hence isolated) cases takes place through attempts to find the individuals potentially infected through epidemiologically relevant contacts. Some individuals found in this way will still be susceptible, some will be latent cases, and others will have developed symptoms. We assume that traced individuals displaying both rash and less specific prodromal fever are isolated an enter the quarantined *Q *class. Traced contacts not displaying any symptoms will be subject to the same observation protocols until they either develop symptoms or conclusively pass the upper limits of the incubation period. We distinguish in the model between susceptible individuals under observation, *O*, and traced cases still in the latent stage, *E*_*T *_.

Tracing takes place across network links in a manner analogous to infection, and this is the reason why models that include tracing must explicitly include network structure. We therefore formulate the model rates in the same manner as for transmission of infection above:(3)

For a given isolated individual who is subject to contact tracing, we assume that each contact in the transmission network is discovered independently with a probability of ∈ = *ρ*/(*ρ *+ *g*_*Q*_) = 0.8 [5]. The mathematical reasoning for this is given in Additional file 1.

#### Vaccination

Smallpox is a vaccine-preventable illness, meaning that susceptible individuals who are successfully vaccinated acquire long-lasting immunity, and in the model are placed in the class of individuals removed due to vaccination, *V*. Our probability of successful vaccination given dose delivery is *ϵ*_1 _= 0.975 [14]. Vaccine can also be given to asymptomatic individuals incubating infection, and in this case there is a significantly reduced probability of success, *ϵ*_2 _= 0.3 [23]. Vaccination is not without its dangers, and we take a baseline probability of mortality following vaccination of *δ*_*V *_= 10*-*5 [24], which we vary during analysis. When only identified contacts of isolated cases are vaccinated, this creates the rules below.(4)

The rate *g*_*O *_= (1/15) days^-1 ^is given by the length of the observation period for observed suspected cases [5].

When we also consider mass vaccination, then we need to introduce the class *S*_*V*_, which represents those susceptibles not contraindicated for vaccination (which we take to be around 70% of the population [25]). When mass vaccination starts, these are then depleted(5)

where *v *is the absolute rate (people per day) at which a population can be vaccinated. The estimation of this quantity is really a question for operational research, and therefore falls outside the scope of this paper. From discussions with the Department of Health, we were able to obtain their estimate that the country could be vaccinated within a week. Since our results are not particularly sensitive to this parameter, the implications of varying it are reasonably clear (slower vaccination is less effective and vice versa), and we do not have access to sufficient information to make an estimate of our own, we make use of the value of one week. *v *is therefore equal to the population of Great Britain not contraindicated for vaccination (70% of 57 million) multiplied by the inverse time required to vaccinate, 1/7 days^-1 ^.

### Spatial scale

One of the main aims of our work is to consider the optimal spatial scale for intervention, and so here we present the data and modelling framework within which such assessments can be made.

#### The NUTS classification

**Figure 2:**
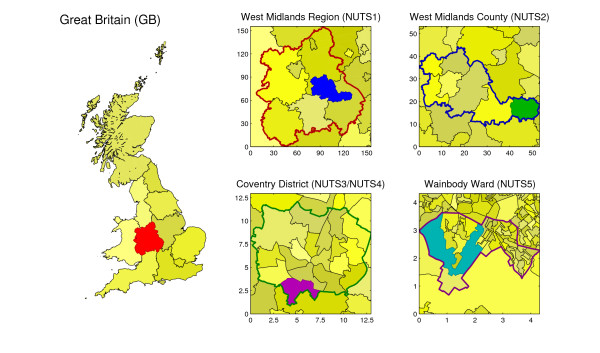
**Range of geographical units around Output Area 00CQFP0009, which contains the main campus of the University of Warwick**. Numbers shown are distances in kilometres

We make use of a hierarchy of statistical units in our analysis. These start at Great Britain (GB), and work down through five Nomenclature of Units for Territorial Statistics (NUTS) levels to the whole of Great Britain. The definition of NUTS regions as currently used by the Office for National Statistics is, at the time of writing, available online at [26], although the NUTS4 and NUTS5 regions relating to 2001 census data have now been replaced by LAU1 and LAU2 regions respectively. Finally we also consider Output Areas, which are very small, local patches with a population of a few hundred people defined for the 2001 census. Figure 2 shows this hierarchy for the regions around the University of Warwick--of particular interest is the common occurrence that NUTS3 and NUTS4 regions are identical around a location. The advantage of this NUTS-based framework is that the highly complex arrangements of different statistical, administrative and historic geographical aggregations in Great Britain can be studied in a systematic manner. It is also likely that decisions to vaccinate (or implement other control measures) will be acted upon at one of these administrative scales. As a point of clarification, although Northern Ireland forms part of the United Kingdom, we deal only with Great Britain since this forms a natural geographically connected epidemiological unit, while Northern Ireland is more strongly connected to the Republic of Ireland [27].

**Figure 3:**
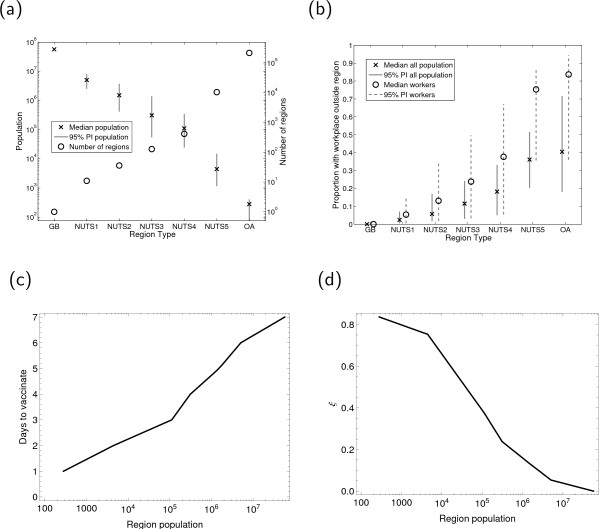
**Characteristics of seven spatial scales in England, Wales and Scotland (Great Britain) and inferred outwardness and time to vaccinate as a function of region population**. (a) shows the median and variability in population size at each scale, together with the number of regions. (b) shows the commuting data at each scale, (c) shows the inferred time to vaccinate against population size, and (d) the inferred outwardness against population size.

We then make use of data on population sizes and commuter movements from the 2001 census [27-30] to make a statistical comparison of these regions as shown in Figure 3. The spread of population sizes, together with median values, at each geographical aggregation is shown in Figure 3(a). We also use census data on home and workplace locations to plot both spreads and medians of both the proportions of workers and the proportions of the total region population that work outside the region at each scale in Figure 3(b). From these two plots, we see that the seven classifications we have chosen cover the full range of population sizes and geographical scales that one would want to consider.

#### Ring vaccination

As in the section above on vaccination, the Department of Health provided us with an estimate of the time to vaccinate a district as three days. We then extrapolate to Figure 3(c) in order to consider multiple spatial scales, although again our results are not highly sensitive to these parameters.

#### Interaction between regions

Our approach to spatial interaction between regions considers disease escape from the region around the initial cases through a tractable model known as a *Poisson process*. Such an approach has also been suggested as a method for model simplification for pandemic influenza [31]. The mathematical description of this process is presented in Additional file 1.

In order to make use of this method, we need to have a measure of the propensity of a region's resident population to leave the area (and hence transfer infection) on a day to day basis. To our knowledge, there is no technical term for this value; we use the term *outwardness *and denote it *ξ*. We then estimate the outwardness of a geographical scale to be the median proportion of workers living in a region at that scale and working outside it (i.e. we assume that non-workers are, in this respect, like workers). We then extrapolate the raw data in Figure 3(b) through linear interpolation on a logarithmic population scale to get the general relationship between population and outwardness shown in Figure 3(d).

The rate at which infection escapes from the region around the initial cases is therefore proportional to the outwardness, and depends on the number if prodromal cases [*P*] and fully symptomatic cases [*I*] as below(6)

Here, we parameterise the significantly reduced movement of symptomatic infectious individuals through multiplying their probability of travel by a factor (1 - *κ*), with the default value of *κ *= 0.9 [5].

### Optimisation of response

Our results consider a six-month outbreak, centred on either a single region or number of regions. For each region, we are able to calculate an expected mortality from the final numbers vaccinated and removed, and the respective mortality risks *δ*_*V *_and *δ*. We are also able to calculate a probability of escape from the region. Logistical and ethical factors become important in the consideration of which outcomes are most desirable, however, and so our approach is to consider a simply described optimal outcome within two release scenarios.

#### Initial cases and detection threshold

Our first assumption concerning initial cases is that they are concentrated, and the epidemic starts with *E*_0 _= 10 latent cases in one area. While in principle this could be significantly higher, the more severe epidemic arising from such a situation may favour mass vaccination as an intervention. We therefore choose to consider an initial outbreak size where there is the strong possibility of containment through case isolation and contact tracing alone.

We assume that until a certain number of fully symptomatic cases has been seen and diagnosed, no public response is instigated. The triggering of interventions is modelled by introducing control measures to the system once [*I*] + [*R*] ≥ *I*_trig_. We take *I*_trig_= 4, for the same reason as our relatively conservative choice of *E*_0 _above.

In the event that secondary areas are infected, we assume that in a situation of heightened awareness and public health response, the secondary epidemic is initialised by  = 1 initial cases and detected once  = 1 symptomatic cases are present.

An alternative scenario is to consider *M*_0 _= 10 maximally dispersed initial cases, each in a distinct region of the size under consideration. In this case, we let the initial regions take 'secondary' parameters; since the outbreaks are simultaneous, this means that the expected national prevalence of clinical infection upon triggering of intervention will be *M*_0_. This is reasonable since dispersed cases are collectively less likely to trigger intervention.

In terms of interpretation, our baseline assumption for regions of relative small sizes would correspond to an accidental/intentional release from a terror cell operating from a domestic address, and at larger sizes a release involving the resident population of a city or region. The dispersed assumption would, on the other hand, involve the other extreme of a release involving a largely transient population of commuters or shoppers. We note that at country level (Great Britain) the dispersed scenario should be interpreted as identical to the NUTS1 level dispersed scenario.

#### Minimisation of expected mortality

The decision about what constitutes an optimal outcome is essentially political, and so our focus is on how epidemiological outputs vary with spatial scale and contact network structure. Nevertheless, we also wish to demonstrate how optimisation can be considered and so also calculate the total expected mortality in primary and secondary affected regions as a simply described and calculated quantity for optimisation. The full details of calculation of this quantity are in Additional file 1.

## Results and Discussion

### Temporal dynamics

**Figure 4:**
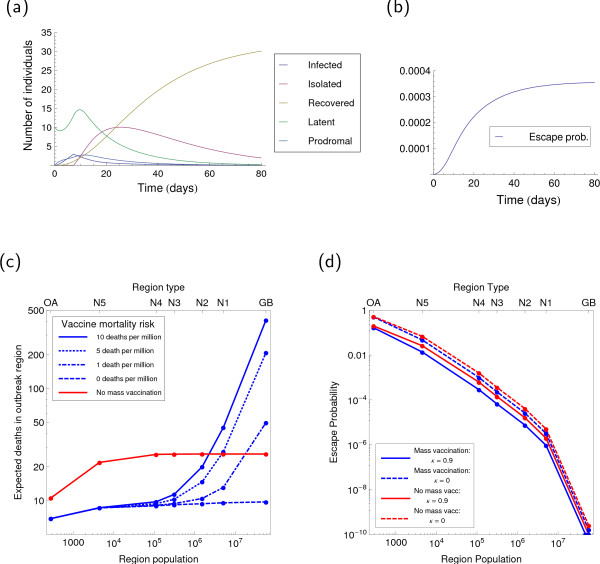
**Baseline dynamics for network smallpox model**. The dynamical variables associated with a free-fall epidemic are shown growing in Pane (a), until the trigger is reached and intervention parameters come to dominate and the outbreak is eventually contained. (b) the cumulative probability of escape grows with untreated cases then levels off. Overall outcomes for the seven spatial scales in England, Wales and Scotland are shown in Panes (c) and (d).

The model's dynamical behaviour, for all default choices in Table 1, is shown in Figure 4(a,b). The 'baseline' values of population size and outwardness are chosen for simple comparison with other published city-level and district-based models, which operate at a similar population size, but these parameters are varied everywhere else in our analysis. Figure 4(a) shows a rise in symptomatic, prodromal and latent infection until case isolation and other interventions are implemented (at approximated 7 days), when the epidemic becomes controlled. However, even after these measures are implemented, the number of latent and isolated individuals continues to rise for several days or weeks. Figure 4(b) shows the total escape probability rising monotonically over time, with a larger gradient at higher prevalence of infection, as would be expected.

### Spatial scale

Using the geographical framework discussed above, varying the vaccine mortality risk and mobility of fully symptomatic cases, but otherwise using the baseline parameters of Table 1, gives the results shown in Figure 4(c,d).

We find, as shown in Figure 4(c), that below a particular population size close to two million (if vaccine-induced mortality *δ*_*V *_is 10 deaths per million vaccinated) local mass vaccination is optimal, with the preferred spatial scale being defined such that there is reasonable confidence that all existing cases are contained at that scale. Above this critical population size, the number of vaccine-induced deaths becomes unacceptably large. Our conclusion is that, even if vaccine-induced mortality is significantly lower, for the small release considered here vaccination of the whole country is likely to be unnecessary given that vaccination at smaller spatial scales and more individually targeted approaches will have brought the epidemic under control.

In terms of escape of infection, Figure 4(d) shows that both the reduced mobility of symptomatic cases and local mass vaccination have a significant impact on final probability. While this is not plotted for clarity, increasing *κ *still further does not have a large effect. The dominant trend is, in any case, that the outwardness of a region is the main determining factor of magnitude of escape probability.

### Parameter sensitivity

**Figure 5:**
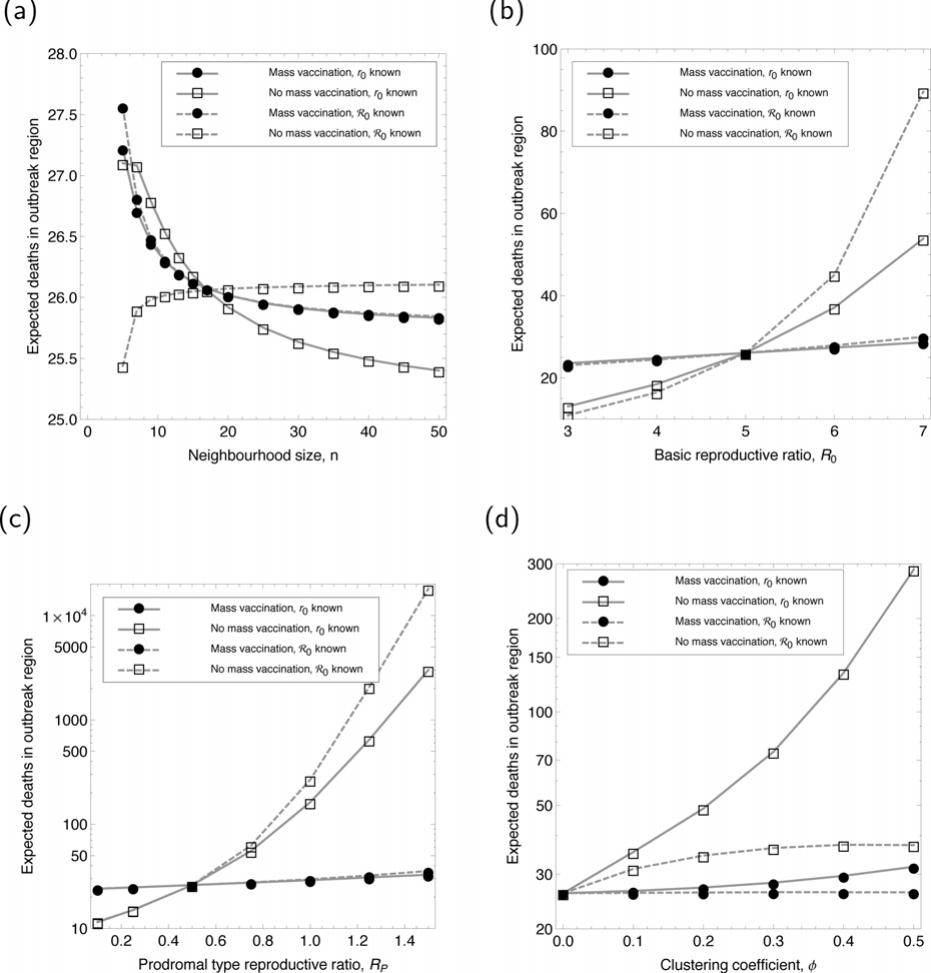
**Effects of varying network and disease parameters on expected deaths**. The effects are shown of modifying (a) neighbourhood size *n*, (b) overall transmission *R*_0_, (c) prodromal transmission *R*_*P*_, and (d) clustering *ϕ*.

To assess the impact of various parameters on the model conclusions, we consider the population size (*N *≈ 2.4 *× *10^6^) at which the expected number of deaths (given *δ*_*V *_= 10^-5^) is the same both with and without local mass vaccination. Using the interpolations shown in Figure 3(c,d), we can then see how modification of a baseline parameter moves us away from the point at which both strategies are equivalent. The results from this analysis are shown for within-region deaths in Figure 5 and for escape probabilities in Additional file 1.

When examining sensitivity to parameter choices, it is important to consider which basic observables of the epidemic should be maintained while the parameters are varied. Here, we have decided to fix the basic reproductive ratio as this is undoubtedly the key observation from any epidemic. For example, as we vary the number of contacts, the transmission rate per contact is varied to compensate for this change. This is analogous to ensuring that all models are fit to the same early epidemic behaviour. One difficulty with fitting to the basic reproductive ratio is that it can be measured in two main ways: either directly by examining the contacts of each infected case, or indirectly by calculating the early growth rate of the epidemic. Unfortunately, for epidemics with complex natural histories and network-based transmission these two methods of calculating the basic reproductive ratio are not in direct agreement, and we therefore show two sets of results in which either measurement is held constant. To make this distinction clear, we denote an individually measured reproduction number ℛ_0 _and one inferred from early growth *r*_0_.

We have found relatively small effects from varying the neighbourhood size *n *at constant *R*_0 _(Figure 5(a)), although varying the reproductive ratio itself has the strong but predictable effect of favouring mass vaccination for larger values of *R*_0 _(Figure 5(b)). What we find significantly more important is the amount of transmission during the prodromal phase of disease, parameterised by *R*_*P *_(Figure 5(c)). While this is highly unlikely to be over 1, a similar effect would be seen from a delay in isolation of infectious individuals, which can be shown in a general manner using threshold arguments [22]. Investigating the effects of clustering (Figure 5(d)) gives strong results, which are both subtle and counter-intuitive, although some theoretical progress has been made in understanding the impact of clustering on contact tracing [6]. In particular, we see in Figure 5(d) that whether *R*_0 _has been estimated from early growth in prevalence or from contact-tracing data has a profound effect on the impact of clustering.

### Optimisation and global sensitivity

**Figure 6:**
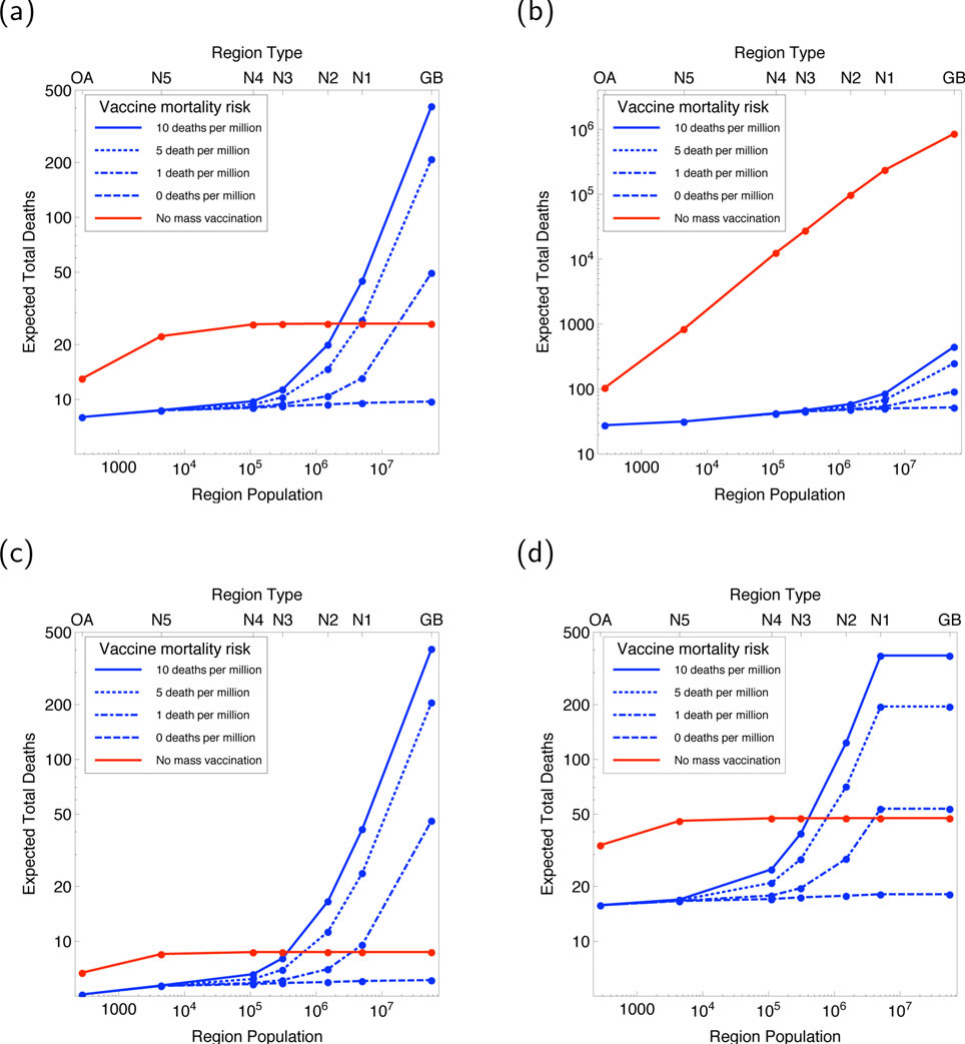
**Outcomes combined into a trade-off**. (a) predicted full deaths for baseline scenario with all cases contained inside one region. (b) 'worst' parameters cause the outbreak to be uncontrolled by targeted interventions, leading to preference for national mass vaccination (the massive increase in overall mortality in this case necessitates a different *y*-axis from other panes). (c) 'best' scenario within the parameters varied changes little. (d) Dispersed trade-off with initial cases maximally dispersed leads to a more severe outbreak but does not qualitatively change the predictions.

The optimisation problem of minimising expected total mortality is addressed in Figure 6. This figure shows the existence of a spatial cutoff above which mass vaccination causes excess total mortality for our baseline parameters in Figure 6(a). Figure 6(b), however, shows that for significantly 'worse' parameters--*R*_0 _= 7, *R*_*P *_= 1.5, *ϕ *= 0.5--this conclusion can be reversed and no such cutoff is seen and mass vaccination is always optimal. Although we do not think such a scenario particularly likely, this points to the importance of real-time estimation in the event of an outbreak. On the other hand, the spatial cutoff scale is reduced for 'better' parameters--*R*_0 _= 3, *R*_*P *_= 0.1, *ϕ *= 0--as shown in Figure 6(c). When we consider the 'dispersed' assumption in Figure 6(d), delayed intervention causes a generally more severe outbreak but does not in itself remove the existence of a population size above which mass vaccination is suboptimal. Since the dispersed scenario has no interpretation when applied to the whole of Great Britain, we simply duplicate the NUTS1 result at this point.

### Parameters not varied

There are a large number of tasks for realistic, informative smallpox modelling, and our particular focus has been on outbreak severity, the impact of contact-network structure, spatial scale and level of undetected transmission. It is worth considering briefly the likely impact of two other extensions to simple models known to be important for general policy conclusions.

Firstly, we have assumed simple rates of transmission between infectious states rather than a more sophisticated class-age structure. Our methodology allows for the inclusion of extra realism of this form through the method of stages, where clinically distinct disease states are broken down into additional sub-compartments. This brings significant extra computational overheads, however when considering a binary choice about vaccination the current understanding of such realism is that it should not qualitatively modify our results, and would be most important if our approach were extended to an accurate system of real-time estimation.

Secondly, we have assumed homogeneity at the individual level, while risks of death, mixing patterns and spatial location are likely to be highly heterogeneous. Again, given that we were only considering mean behaviour of the system this does not invalidate our approach, but would be important if policy decisions needed to be calibrated to 'reasonable worst case' rather than expected outcomes.

## Conclusions

In dealing with the optimisation of public-health response to deliberate release of smallpox, we are considering a highly complicated system that is not directly amenable to experimental testing. This means that there will always be a degree of uncertainty associated with conclusions presented, however strongly these manifest themselves in a model.

Despite this general caveat, investigation of the problem for Great Britain suggests that, for a wide variety of parameter choices, and with differing modelling approaches, a vaccination strategy that involves a wider section of the population than the traced contacts of isolated cases but one that stops short of vaccinating the whole country is likely to be optimal. Such a conclusion is, we believe, likely to be robust in the event of a relatively small initial outbreak and given our best estimates of the contagiousness and natural history of *variola*. It could be overturned for a particularly virulent or large outbreak, however similar measures were taken during the eradication programme and were effective in controlling historical outbreaks [32]. Additionally, a larger outbreak may be observed sooner, leading to earlier detection and isolation of cases. Another key message is that delays in case isolation, or equivalently more transmission in the prodromal phase than was previously observed, are extremely significant, and that slow response has the potential to present the most significant barrier to the efficacy of non-pharmaceutical interventions. Conversely, there are significant advantages to faster case isolation and treatment.

We have generalised on previous work in two main ways. Firstly, we have extended the treatment of contact networks to include the realistic assumption of clustering, which has an extremely important impact on the efficacy of contact tracing. However, the measurement of even basic quantities like the clustering coefficient *ϕ *for epidemiologically relevant contacts has yet to be attempted at a large geographical scale, particularly in non-household contexts, and we suggest that this could be an important measurement to make given its substantial effect on predicted outcomes.

Secondly, we have considered human movement patterns across a full range of geographical scales, enabling the calculation of an optimal scale for intervention. Furthermore, the techniques in this paper can be used to analyse situations where the numbers people leaving an area are inferable but the full region to region 'commuting' behaviour is unknown. As such this could be applied to other countries that don't collect detailed workplace location information in their census programmes.

There are many further generalisations of our work that could be considered, which would be of use regarding a response to a wide variety of emerging infectious agents. In particular, the inclusion of higher-order clusters in human contact networks, such as households, workplaces and social groups, is likely to be of significant importance. Also likely to be significant is the modification of 'baseline' patterns of movement and social interaction in response to perceived risk of infection. Consideration should also be made of the logistical constraints on local- and national-scale policies. We hope that the current work presents a useful foundation for the consideration of these and other questions.

## Competing interests

The authors declare that they have no competing interests.

## Authors' contributions

TH carried out the mathematical formulation of the model and coding. IH, LD and MJK provided parameter values, and informed the choice of simulations to undertake. All authors contributed to the writing of the paper.

## Pre-publication history

The pre-publication history for this paper can be accessed here:

http://www.biomedcentral.com/1471-2334/10/25/prepub

## Supplementary Material

Additional file 1 **Supplementary Material**. We have included a supplementary PDF containing mathematical and simulation results necessary to reproduce our work but not necessary for the main thrust of argument in the paper. This can be viewed in a free viewer such as Adobe Acrobat Reader. Click here for file
