# Supplementary material for: Contingency planning for a deliberate release of smallpox in Great Britain - the role of geographical scale and contact structure
Source: BMC Infect Dis. 2010 Feb 14;10:25. doi: 10.1186/1471-2334-10-25 (PMC2831898; doi:10.1186/1471-2334-10-25)
Supplement: Additional file 1 — Supplementary Material. We have included a supplementary PDF containing mathematical and simulation results necessary to reproduce our work but not necessary for the main thrust of argument in the paper. This can be viewed in a free viewer such as Adobe Acrobat Reader. [file 1471-2334-10-25-S1.PDF]

Contingency planning for a deliberate release of smallpox in Great Britain—the role of geographical scale and contact structure:

# Supplementary Material

Thomas House, Ian Hall, Leon Danon and Matt J. Keeling

This supplementary material consists of three sections: firstly, we present the formal mathematical underpinnings of our work, which are necessary for reproduction of the results; secondly, we present a supplementary figure of sensitivity analysis; and finally, we present the full system of differential equations that defines our model.

# 1 Mathematical model

## 1.1 Probability of a network event

Suppose we have a network node in a state $\zeta$, from which it 'recovers' at rate $g$, and which 'transmits' its state to a neighbour currently in state $\gamma$ at a rate $\tau$. We then define

$$\begin{aligned} p(t) &:= \text{Pr(Transmission happened at some time t}' \leq \text{t)} \ , \\ q(t) &:= \text{Pr(The system remains in the state } \zeta \leftrightarrow \gamma \text{ at time t)} \ . \end{aligned} \tag{1}$$

This system is then described by the equations

$$\begin{aligned} \frac{dq}{dt} &= -(\tau + g)q(t) \ , \\ \frac{dp}{dt} &= \tau q(t) \ . \end{aligned} \tag{2}$$

This has the solution

$$q(t) = e^{-(\tau+g)t} \ , \quad p(t) = \frac{\tau}{\tau + g}\left(1 - e^{-(\tau+g)t}\right) \ . \tag{3}$$

So clearly, the final probability of transmission is $\tau/(\tau + g)$ and the expected number of transmission is

$$\langle \text{number of transmissions} \rangle = n\frac{\tau}{\tau + g} \ , \tag{4}$$

for a node with $n$ links.

## 1.2 Keeping $R_0, R_P$ constant

We consider two ways to fit our network rates to reproduction numbers. For the first of these, we define the relevant type reproduction numbers through the 'early growth' of the system, defined through linearisation of the pairwise system using the following Ansatz:

$$
\begin{aligned}
[\dot{I}] &= \mathcal{K}[I] , \\
[A] &= [A]_{t=0} + k_A[I] , \\
[AB] &= [AB]_{t=0} + k_{AB}[I] .
\end{aligned} \tag{5}
$$

We then define

$$
r_I := \frac{\tau_I k_{IS}}{g_I} , \qquad r_P := \frac{\tau_I k_{PS}}{g_P} , \qquad r_0 := r_I + r_P . \tag{6}
$$

These 'early growth' reproduction numbers can then be set to the desired values for $R_0, R_P$. Substituting these together with the linear Ansatz and ignoring terms of order $I(t)^2$ and higher allows us to fix $\tau_I, \tau_P$. It is worth noting that during the early part of an epidemic, the growth of all disease compartments is governed by just one Malthusian parameter, and so $r_I$ and $r_P$ should be interpreted as an natural, approximate apportionment of population-level disease growth to each disease class rather than a rigorous result for prodromal and infectious reproduction numbers on networks.

An alternative approach is to hold constant the literal expected number of secondary infections created by a single infectious individual in a fully susceptible population (the textbook definition of $R_0$), which we write as

$$
\mathcal{R}_I := \frac{n\tau_I}{\tau_I + g_I} , \qquad \mathcal{R}_P := \frac{n\tau_P}{\tau_P + g_P} , \qquad \mathcal{R}_0 := \mathcal{R}_I + \mathcal{R}_P . \tag{7}
$$

The reasoning behind these definitions is expounded in Section 1.1 above. We note that contact network structure will modify (7) after the initial generation, however any individual-level measurement in the release scenario we are considering will almost certainly still be before this modification.

## 1.3 Improved closure methods

The unclosed pairwise equations, generated automatically from rules using Mathematica 6.0 code, are included in section 3 below. To integrate these, approximations linking triples and pairs are needed.

Our code relies on a novel method for closure of triples. Where the prevalence of $[AB]$ is being modified by the action of a $C$ upon $B$, the following approximation significantly improves the behaviour of the pairwise system during numerical integration:

$$
[ABC] \approx (n-1)\left((1-\phi)\frac{[AB][BC]}{n[B]} + \phi\frac{[AB][BC][CA]}{[A]\sum_a([aB][aC]/[a])}\right) . \tag{8}
$$

We found that even sophisticated software such as Mathematica, using adaptive algorithms for ODE integration and a working precision of 50, gave unacceptable performance for the sheer number and complexity of equations involved using standard closure. This problem was avoided for the improved closure above.

## 1.4 Escape from control region

If we suppose that the control region exerts an external force of infection, $f(t)$, over time, then the probability of escape over time, $h(t)$, is given in terms of this force of infection by

$$\dot{h}(t) = (1 - h(t))\, f(t)\ . \tag{9}$$

This model is generally tractable in the sense that (9) can be integrated directly to give

$$h(t) = 1 - e^{-\int_0^t f(u)du}\ , \tag{10}$$

however we need a full expression for the force of infection, which is

$$f(t) = \frac{n\xi}{N}\left(\tau_P\,[P] + (1-\kappa)\tau_I\,[I]\right)\ . \tag{11}$$

## 1.5 Trade-off for optimisation

We start by making the following definitions for final escape probability, attack rate and proportion vaccinated in $m$-ary affected regions:

$$h_\infty^{(m)} := \lim_{t\to\infty} h(t,m)\ , \quad R_\infty^{(m)} := \lim_{t\to\infty}[R]_{t,m}\ , \quad V_\infty^{(m)} := \lim_{t\to\infty}[V]_{t,m}\ . \tag{12}$$

The expected total deaths from an outbreak in one region is then

$$T := R_\infty^{(1)}\delta + V_\infty^{(1)}\delta_V + M\left(1 - (1-h_\infty)^{1/M}\right)\left(R_\infty^{(2)}\delta + V_\infty^{(2)}\delta_V\right)\ , \tag{13}$$

where $M$ is the number of other regions of the same type as the initial outbreak region, shown in Figure 3(a) of the main text. $R_\infty^{(2)}$ and $V_\infty^{(2)}$ are calculated by modifying both $I_{\text{trig}}$ and $E_0$ to one case, as in Table 1 of the main text. The equivalent expression for a dispersed outbreak is

$$T_d := \left(M_0 + \max(M+1-M_0, 0)\left(1 - (1-h_\infty)^{1/(M+1-M_0)}\right)\right)\left(R_\infty^{(2)}\delta + V_\infty^{(2)}\delta_V\right)\ . \tag{14}$$

## 2 Supplementary figure

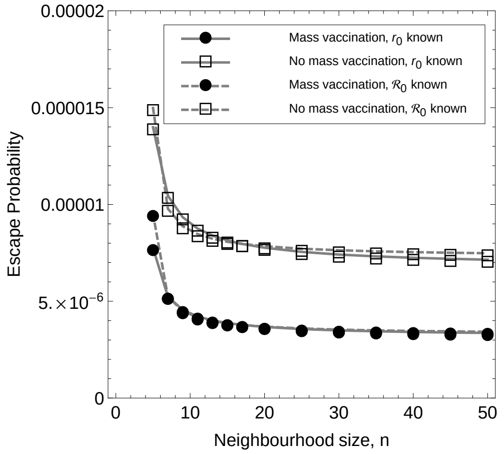


(a) Varying $n$

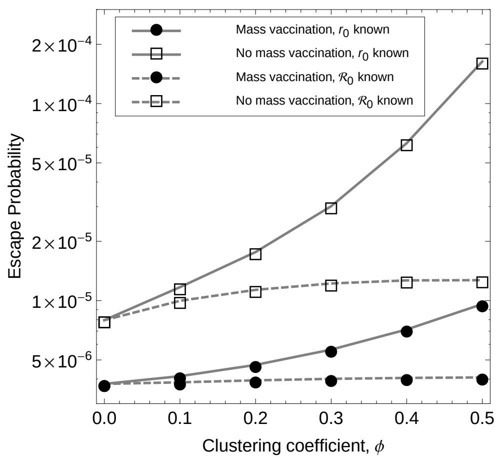


(b) Varying $\phi$

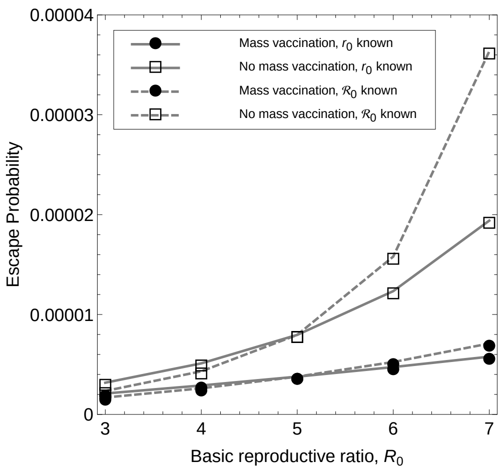


(c) Varying $R_0$

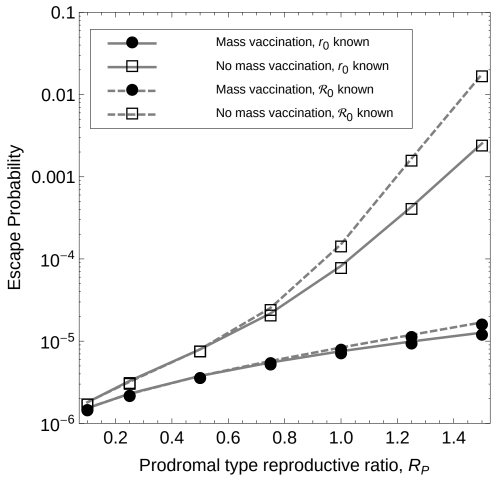


(d) Varying $R_P$

Figure 1: Effects of varying network and disease parameters on cumulative escape probability.

## 3 Complete system of unclosed ODEs

$$
\begin{aligned}
[E]_0 &= E_0 \\
[S]_0 &= (N - E_0)(1-\gamma) \\
[S_V]_0 &= (N - E_0)\gamma \\
[EE]_0 &= \frac{E_0^2 n}{N} \\
[ES]_0 &= \frac{E_0 n(N - E_0)(1-\gamma)}{N} \\
[ES_V]_0 &= \frac{E_0 n(N - E_0)\gamma}{N} \\
[SE]_0 &= \frac{E_0 n(N - E_0)(1-\gamma)}{N} \\
[SS]_0 &= \frac{n(N - E_0)^2(1-\gamma)^2}{N} \\
[SS_V]_0 &= \frac{n(N - E_0)^2(1-\gamma)\gamma}{N} \\
[S_V E]_0 &= \frac{E_0 n(N - E_0)\gamma}{N} \\
[S_V S]_0 &= \frac{n(N - E_0)^2(1-\gamma)\gamma}{N} \\
[S_V S_V]_0 &= \frac{n(N - E_0)^2\gamma^2}{N} \\
[\dot{E}] &= -g_E[E] - \rho[EQ] + \tau_I[SI] \\
&\quad + \tau_P[SP] + \tau_I[S_V I] + \tau_P[S_V P] \\
[\dot{E_T}] &= -g_E(1-\epsilon_2)[E_T] - g_E\epsilon_2[E_T] \\
&\quad + \rho[EQ] \\
[\dot{I}] &= -g_I[I] + g_P(1-\theta)[P] \\
&\quad - \rho[IQ] \\
[\dot{O}] &= -g_O(1-\epsilon_1)[O] - g_O\epsilon_1[O] \\
&\quad + \rho[SQ] + \rho[S_V Q]
\end{aligned}
$$

$$
\begin{aligned}
[\dot{P}] &= g_E[E] - g_P(1-\theta)[P] - \rho[PQ] \\
&\quad - g_P\theta[P]\Theta([I]+[R]-I_{\text{trig}}) \\
[\dot{Q}] &= g_E(1-\epsilon_2)[E_T] - g_Q[Q] + \rho[IQ] \\
&\quad + \rho[PQ] + g_P\theta[P]\Theta([I]+[R]-I_{\text{trig}}) \\
[\dot{R}] &= g_I[I] + g_Q[Q] \\
[\dot{S}] &= g_O(1-\epsilon_1)[O] - \tau_I[SI] - \tau_P[SP] \\
&\quad - \rho[SQ] \\
[\dot{S_V}] &= -\tau_I[S_VI] - \tau_P[S_VP] - \rho[S_VQ] \\
&\quad - \nu\Theta([I]+[R]-I_{\text{trig}})\Theta([S_V]) \\
[\dot{V}] &= g_E\epsilon_2[E_T] + g_O\epsilon_1[O] + \nu\Theta([I]+[R]-I_{\text{trig}})\Theta([S_V]) \\
[\dot{EE}] &= -2g_E[EE] - 2\rho[EEQ] + 2\tau_I[ESI] \\
&\quad + 2\tau_P[ESP] + 2\tau_I[ES_VI] + 2\tau_P[ES_VP] \\
[\dot{EE_T}] &= -g_E[EE_T] - g_E(1-\epsilon_2)[EE_T] \\
&\quad - g_E\epsilon_2[EE_T] + \rho[EEQ] - \rho[E_TEQ] + \tau_I[E_TSI] \\
&\quad + \tau_P[E_TSP] + \tau_I[E_TS_VI] + \tau_P[E_TS_VP] \\
[\dot{EI}] &= -g_E[EI] - g_I[EI] + g_P(1-\theta)[EP] \\
&\quad + \tau_I[SI] + \tau_I[S_VI] - \rho[EIQ] - \rho[IEQ] \\
&\quad + \tau_I[ISI] + \tau_P[ISP] + \tau_I[IS_VI] + \tau_P[IS_VP] \\
[\dot{EO}] &= -g_E[EO] - g_O(1-\epsilon_1)[EO] \\
&\quad - g_O\epsilon_1[EO] + \rho[ESQ] + \rho[ES_VQ] - \rho[OEQ] \\
&\quad + \tau_I[OSI] + \tau_P[OSP] + \tau_I[OS_VI] + \tau_P[OS_VP] \\
[\dot{EP}] &= g_E[EE] - g_E[EP] - g_P(1-\theta)[EP] \\
&\quad + \tau_P[SP] + \tau_P[S_VP] - \rho[EPQ] - \rho[PEQ] \\
&\quad + \tau_I[PSI] + \tau_P[PSP] + \tau_I[PS_VI] + \tau_P[PS_VP] \\
&\quad - g_P\theta[EP]\Theta([I]+[R]-I_{\text{trig}})
\end{aligned}
$$

$$
\begin{aligned}
\dot{[EQ]} &= g_E(1-\epsilon_2)[EE_T] - g_E[EQ] - g_Q[EQ] \\
&\quad - \rho[EQ] + \rho[EIQ] + \rho[EPQ] - \rho[QEQ] \\
&\quad + \tau_I[QSI] + \tau_P[QSP] + \tau_I[QS_VI] + \tau_P[QS_VP] \\
&\quad + g_P\theta[EP]\Theta([I]+[R]-I_{\mathrm{trig}}) \\
\dot{[ER]} &= g_I[EI] + g_Q[EQ] - g_E[ER] - \rho[REQ] \\
&\quad + \tau_I[RSI] + \tau_P[RSP] + \tau_I[RS_VI] + \tau_P[RS_VP] \\
\dot{[ES]} &= g_O(1-\epsilon_1)[EO] - g_E[ES] - \tau_I[ESI] \\
&\quad - \tau_P[ESP] - \rho[ESQ] - \rho[SEQ] + \tau_I[SSI] \\
&\quad + \tau_P[SSP] + \tau_I[SS_VI] + \tau_P[SS_VP] \\
\dot{[ES_V]} &= -g_E[ES_V] - \frac{\nu}{[S_V]}\Theta([I]+[R]-I_{\mathrm{trig}})\Theta([S_V])[ES_V] \\
&\quad - \tau_I[ES_VI] - \tau_P[ES_VP] - \rho[ES_VQ] - \rho[S_VEQ] \\
&\quad + \tau_I[S_VSI] + \tau_P[S_VSP] + \tau_I[S_VS_VI] + \tau_P[S_VS_VP] \\
\dot{[EV]} &= g_E\epsilon_2[EE_T] + g_O\epsilon_1[EO] - g_E[EV] - \rho[VEQ] \\
&\quad + \tau_I[VSI] + \tau_P[VSP] + \tau_I[VS_VI] + \tau_P[VS_VP] \\
&\quad + \frac{\nu}{[S_V]}[ES_V]\Theta([I]+[R]-I_{\mathrm{trig}})\Theta([S_V]) \\
\dot{[E_TE]} &= -g_E[E_TE] - g_E(1-\epsilon_2)[E_TE] \\
&\quad - g_E\epsilon_2[E_TE] + \rho[EEQ] - \rho[E_TEQ] + \tau_I[E_TSI] \\
&\quad + \tau_P[E_TSP] + \tau_I[E_TS_VI] + \tau_P[E_TS_VP] \\
\dot{[E_TE_T]} &= -2g_E(1-\epsilon_2)[E_TE_T] - 2g_E\epsilon_2[E_TE_T] \\
&\quad + 2\rho[E_TEQ] \\
\dot{[E_TI]} &= -g_I[E_TI] - g_E(1-\epsilon_2)[E_TI] \\
&\quad - g_E\epsilon_2[E_TI] + g_P(1-\theta)[E_TP] - \rho[E_TIQ] \\
&\quad + \rho[IEQ]
\end{aligned}
$$

$$
\begin{aligned}
[E_T\dot{O}] = & -g_O(1-\epsilon_1)[E_TO] - g_O\epsilon_1[E_TO] \\
& - g_E(1-\epsilon_2)[E_TO] - g_E\epsilon_2[E_TO] + \rho[E_TSQ] \\
& + \rho[E_TS_VQ] + \rho[OEQ] \\
[E_T\dot{P}] = & \, g_E[E_TE] - g_E(1-\epsilon_2)[E_TP] - g_E\epsilon_2[E_TP] \\
& - g_P(1-\theta)[E_TP] - \rho[E_TPQ] + \rho[PEQ] \\
& - g_P\theta[E_TP]\Theta([I]+[R]-I_{\mathrm{trig}}) \\
[E_T\dot{Q}] = & \, \rho[EQ] + g_E(1-\epsilon_2)[E_TE_T] - g_Q[E_TQ] \\
& - g_E(1-\epsilon_2)[E_TQ] - g_E\epsilon_2[E_TQ] + \rho[E_TIQ] \\
& + \rho[E_TPQ] + \rho[QEQ] + g_P\theta[E_TP]\Theta([I]+[R]-I_{\mathrm{trig}}) \\
[E_T\dot{R}] = & \, g_I[E_TI] + g_Q[E_TQ] - g_E(1-\epsilon_2)[E_TR] \\
& - g_E\epsilon_2[E_TR] + \rho[REQ] \\
[E_T\dot{S}] = & \, g_O(1-\epsilon_1)[E_TO] - g_E(1-\epsilon_2)[E_TS] \\
& - g_E\epsilon_2[E_TS] - \tau_I[E_TSI] - \tau_P[E_TSP] - \rho[E_TSQ] \\
& + \rho[SEQ] \\
[E_T\dot{S}_V] = & -g_E(1-\epsilon_2)[E_TS_V] - g_E\epsilon_2[E_TS_V] \\
& - \frac{\nu}{[S_V]}\Theta([I]+[R]-I_{\mathrm{trig}})\Theta([S_V])[E_TS_V] - \tau_I[E_TS_VI] \\
& - \tau_P[E_TS_VP] - \rho[E_TS_VQ] + \rho[S_VEQ] \\
[E_T\dot{V}] = & \, g_E\epsilon_2[E_TE_T] + g_O\epsilon_1[E_TO] - g_E(1-\epsilon_2)[E_TV] \\
& - g_E\epsilon_2[E_TV] + \rho[VEQ] + \frac{\nu}{[S_V]}[E_TS_V]\Theta([I]+[R]-I_{\mathrm{trig}})\Theta([S_V]) \\
[\dot{IE}] = & -g_E[IE] - g_I[IE] + \tau_I[IS] \\
& + \tau_I[IS_V] + g_P(1-\theta)[PE] - \rho[EIQ] \\
& - \rho[IEQ] + \tau_I[ISI] + \tau_P[ISP] + \tau_I[IS_VI] \\
& + \tau_P[IS_VP]
\end{aligned}
$$

$$
\begin{aligned}
[I\dot{E}_T] =& -g_I[IE_T] - g_E(1-\epsilon_2)[IE_T] \\
& - g_E\epsilon_2[IE_T] + g_P(1-\theta)[PE_T] - \rho[E_T IQ] \\
& + \rho[IEQ] \\
[\dot{II}] =& -2g_I[II] + g_P(1-\theta)[IP] \\
& + g_P(1-\theta)[PI] - 2\rho[IIQ] \\
[I\dot{O}] =& -g_I[IO] - g_O(1-\epsilon_1)[IO] \\
& - g_O\epsilon_1[IO] + g_P(1-\theta)[PO] + \rho[ISQ] \\
& + \rho[IS_V Q] - \rho[OIQ] \\
[I\dot{P}] =& \, g_E[IE] - g_I[IP] - g_P(1-\theta)[IP] \\
& + g_P(1-\theta)[PP] - \rho[IPQ] - \rho[PIQ] \\
& - g_P\theta[IP]\Theta([I] + [R] - I_{\text{trig}}) \\
[I\dot{Q}] =& \, g_E(1-\epsilon_2)[IE_T] - g_I[IQ] - g_Q[IQ] \\
& - \rho[IQ] + g_P(1-\theta)[PQ] + \rho[IIQ] \\
& + \rho[IPQ] - \rho[QIQ] + g_P\theta[IP]\Theta([I] + [R] - I_{\text{trig}}) \\
[I\dot{R}] =& \, g_I[II] + g_Q[IQ] - g_I[IR] + g_P(1-\theta)[PR] \\
& - \rho[RIQ] \\
[I\dot{S}] =& \, g_O(1-\epsilon_1)[IO] - g_I[IS] - \tau_I[IS] \\
& + g_P(1-\theta)[PS] - \tau_I[ISI] - \tau_P[ISP] \\
& - \rho[ISQ] - \rho[SIQ] \\
[I\dot{S}_V] =& -g_I[IS_V] - \tau_I[IS_V] - \frac{\nu}{[S_V]}\Theta([I] + [R] - I_{\text{trig}})\Theta([S_V])[IS_V] \\
& + g_P(1-\theta)[PS_V] - \tau_I[IS_V I] - \tau_P[IS_V P] \\
& - \rho[IS_V Q] - \rho[S_V IQ]
\end{aligned}
$$

$$
\begin{aligned}
[\dot{IV}] &= g_E\epsilon_2[IE_T] + g_O\epsilon_1[IO] - g_I[IV] + g_P(1-\theta)[PV] \\
&\quad - \rho[VIQ] + \frac{\nu}{[S_V]}[IS_V]\Theta([I]+[R]-I_{\text{trig}})\Theta([S_V]) \\
[\dot{OE}] &= -g_E[OE] - g_O(1-\epsilon_1)[OE] \\
&\quad - g_O\epsilon_1[OE] + \rho[ESQ] + \rho[ES_VQ] - \rho[OEQ] \\
&\quad + \tau_I[OSI] + \tau_P[OSP] + \tau_I[OS_VI] + \tau_P[OS_VP] \\
[\dot{OE_T}] &= -g_O(1-\epsilon_1)[OE_T] - g_O\epsilon_1[OE_T] \\
&\quad - g_E(1-\epsilon_2)[OE_T] - g_E\epsilon_2[OE_T] + \rho[E_TSQ] \\
&\quad + \rho[E_TS_VQ] + \rho[OEQ] \\
[\dot{OI}] &= -g_I[OI] - g_O(1-\epsilon_1)[OI] \\
&\quad - g_O\epsilon_1[OI] + g_P(1-\theta)[OP] + \rho[ISQ] \\
&\quad + \rho[IS_VQ] - \rho[OIQ] \\
[\dot{OO}] &= -2g_O(1-\epsilon_1)[OO] - 2g_O\epsilon_1[OO] \\
&\quad + 2\rho[OSQ] + 2\rho[OS_VQ] \\
[\dot{OP}] &= g_E[OE] - g_O(1-\epsilon_1)[OP] - g_O\epsilon_1[OP] \\
&\quad - g_P(1-\theta)[OP] - \rho[OPQ] + \rho[PSQ] \\
&\quad + \rho[PS_VQ] - g_P\theta[OP]\Theta([I]+[R]-I_{\text{trig}}) \\
[\dot{OQ}] &= g_E(1-\epsilon_2)[OE_T] - g_Q[OQ] - g_O(1-\epsilon_1)[OQ] \\
&\quad - g_O\epsilon_1[OQ] + \rho[SQ] + \rho[S_VQ] + \rho[OIQ] \\
&\quad + \rho[OPQ] + \rho[QSQ] + \rho[QS_VQ] + g_P\theta[OP]\Theta([I]+[R]-I_{\text{trig}}) \\
[\dot{OR}] &= g_I[OI] + g_Q[OQ] - g_O(1-\epsilon_1)[OR] \\
&\quad - g_O\epsilon_1[OR] + \rho[RSQ] + \rho[RS_VQ] \\
[\dot{OS}] &= g_O(1-\epsilon_1)[OO] - g_O(1-\epsilon_1)[OS] \\
&\quad - g_O\epsilon_1[OS] - \tau_I[OSI] - \tau_P[OSP] - \rho[OSQ] \\
&\quad + \rho[SSQ] + \rho[SS_VQ]
\end{aligned}
$$

$$
\begin{aligned}
[\dot{OS_V}] =& -g_O(1-\epsilon_1)[OS_V] - g_O\epsilon_1[OS_V] \\
& - \frac{\nu}{[S_V]}\Theta([I]+[R]-I_{\rm trig})\Theta([S_V])[OS_V] - \tau_I[OS_VI] \\
& - \tau_P[OS_VP] - \rho[OS_VQ] + \rho[S_VSQ] + \rho[S_VS_VQ] \\
[\dot{OV}] =& \, g_E\epsilon_2[OE_T] + g_O\epsilon_1[OO] - g_O(1-\epsilon_1)[OV] \\
& - g_O\epsilon_1[OV] + \rho[VSQ] + \rho[VS_VQ] + \frac{\nu}{[S_V]}[OS_V]\Theta([I]+[R]-I_{\rm trig})\Theta([S_V]) \\
[\dot{PE}] =& \, g_E[EE] - g_E[PE] - g_P(1-\theta)[PE] \\
& + \tau_P[PS] + \tau_P[PS_V] - \rho[EPQ] - \rho[PEQ] \\
& + \tau_I[PSI] + \tau_P[PSP] + \tau_I[PS_VI] + \tau_P[PS_VP] \\
& - g_P\theta[PE]\Theta([I]+[R]-I_{\rm trig}) \\
[\dot{PE_T}] =& \, g_E[EE_T] - g_E(1-\epsilon_2)[PE_T] - g_E\epsilon_2[PE_T] \\
& - g_P(1-\theta)[PE_T] - \rho[E_TPQ] + \rho[PEQ] \\
& - g_P\theta[PE_T]\Theta([I]+[R]-I_{\rm trig}) \\
[\dot{PI}] =& \, g_E[EI] - g_I[PI] - g_P(1-\theta)[PI] \\
& + g_P(1-\theta)[PP] - \rho[IPQ] - \rho[PIQ] \\
& - g_P\theta[PI]\Theta([I]+[R]-I_{\rm trig}) \\
[\dot{PO}] =& \, g_E[EO] - g_O(1-\epsilon_1)[PO] - g_O\epsilon_1[PO] \\
& - g_P(1-\theta)[PO] - \rho[OPQ] + \rho[PSQ] \\
& + \rho[PS_VQ] - g_P\theta[PO]\Theta([I]+[R]-I_{\rm trig}) \\
[\dot{PP}] =& \, g_E[EP] + g_E[PE] - 2g_P(1-\theta)[PP] \\
& - 2\rho[PPQ] - 2g_P\theta[PP]\Theta([I]+[R]-I_{\rm trig}) \\
[\dot{PQ}] =& \, g_E[EQ] + g_E(1-\epsilon_2)[PE_T] - g_Q[PQ] \\
& - \rho[PQ] - g_P(1-\theta)[PQ] + \rho[PIQ] \\
& + \rho[PPQ] - \rho[QPQ] + g_P\theta[PP]\Theta([I]+[R]-I_{\rm trig}) \\
& - g_P\theta[PQ]\Theta([I]+[R]-I_{\rm trig})
\end{aligned}
$$

$$
\begin{aligned}
[\dot{PR}] &= g_E[ER] + g_I[PI] + g_Q[PQ] - g_P(1-\theta)[PR] \\
&\quad - \rho[RPQ] - g_P\theta[PR]\Theta([I] + [R] - I_{\text{trig}}) \\
[\dot{PS}] &= g_E[ES] + g_O(1-\epsilon_1)[PO] - \tau_P[PS] \\
&\quad - g_P(1-\theta)[PS] - \tau_I[PSI] - \tau_P[PSP] \\
&\quad - \rho[PSQ] - \rho[SPQ] - g_P\theta[PS]\Theta([I] + [R] - I_{\text{trig}}) \\
[\dot{PS}_V] &= g_E[ES_V] - \tau_P[PS_V] - g_P(1-\theta)[PS_V] \\
&\quad - \tau_I[PS_VI] - \tau_P[PS_VP] - \rho[PS_VQ] - \rho[S_VPQ] \\
&\quad - g_P\theta[PS_V]\Theta([I] + [R] - I_{\text{trig}}) - \frac{\nu}{[S_V]}[PS_V]\Theta([I] + [R] - I_{\text{trig}})\Theta([S_V]) \\
[\dot{PV}] &= g_E[EV] + g_E\epsilon_2[PE_T] + g_O\epsilon_1[PO] - g_P(1-\theta)[PV] \\
&\quad - \rho[VPQ] - g_P\theta[PV]\Theta([I] + [R] - I_{\text{trig}}) + \frac{\nu}{[S_V]}[PS_V]\Theta([I] + [R] - I_{\text{trig}})\Theta([S_V]) \\
[\dot{QE}] &= g_E(1-\epsilon_2)[E_TE] - g_E[QE] - g_Q[QE] \\
&\quad - \rho[QE] + \rho[EIQ] + \rho[EPQ] - \rho[QEQ] \\
&\quad + \tau_I[QSI] + \tau_P[QSP] + \tau_I[QS_VI] + \tau_P[QS_VP] \\
&\quad + g_P\theta[PE]\Theta([I] + [R] - I_{\text{trig}}) \\
[\dot{QE}_T] &= g_E(1-\epsilon_2)[E_TE_T] + \rho[QE] - g_Q[QE_T] \\
&\quad - g_E(1-\epsilon_2)[QE_T] - g_E\epsilon_2[QE_T] + \rho[E_TIQ] \\
&\quad + \rho[E_TPQ] + \rho[QEQ] + g_P\theta[PE_T]\Theta([I] + [R] - I_{\text{trig}}) \\
[\dot{QI}] &= g_E(1-\epsilon_2)[E_TI] - g_I[QI] - g_Q[QI] \\
&\quad - \rho[QI] + g_P(1-\theta)[QP] + \rho[IIQ] \\
&\quad + \rho[IPQ] - \rho[QIQ] + g_P\theta[PI]\Theta([I] + [R] - I_{\text{trig}}) \\
[\dot{QO}] &= g_E(1-\epsilon_2)[E_TO] - g_Q[QO] - g_O(1-\epsilon_1)[QO] \\
&\quad - g_O\epsilon_1[QO] + \rho[QS] + \rho[QS_V] + \rho[OIQ] \\
&\quad + \rho[OPQ] + \rho[QSQ] + \rho[QS_VQ] + g_P\theta[PO]\Theta([I] + [R] - I_{\text{trig}})
\end{aligned}
$$

$$
\begin{aligned}
[\dot{QP}] &= g_E(1-\epsilon_2)[E_T P] + g_E[QE] - g_Q[QP] \\
&\quad - \rho[QP] - g_P(1-\theta)[QP] + \rho[PIQ] \\
&\quad + \rho[PPQ] - \rho[QPQ] + g_P\theta[PP]\Theta([I]+[R]-I_{\mathrm{trig}}) \\
&\quad - g_P\theta[QP]\Theta([I]+[R]-I_{\mathrm{trig}}) \\
[\dot{QQ}] &= g_E(1-\epsilon_2)[E_T Q] + \rho[IQ] + \rho[PQ] \\
&\quad + g_E(1-\epsilon_2)[QE_T] + \rho[QI] + \rho[QP] \\
&\quad - 2g_Q[QQ] + 2\rho[QIQ] + 2\rho[QPQ] + g_P\theta[PQ]\Theta([I]+[R]-I_{\mathrm{trig}}) \\
&\quad + g_P\theta[QP]\Theta([I]+[R]-I_{\mathrm{trig}}) \\
[\dot{QR}] &= g_E(1-\epsilon_2)[E_T R] + g_I[QI] + g_Q[QQ] \\
&\quad - g_Q[QR] + \rho[RIQ] + \rho[RPQ] + g_P\theta[PR]\Theta([I]+[R]-I_{\mathrm{trig}}) \\
[\dot{QS}] &= g_E(1-\epsilon_2)[E_T S] + g_O(1-\epsilon_1)[QO] \\
&\quad - g_Q[QS] - \rho[QS] - \tau_I[QSI] - \tau_P[QSP] \\
&\quad - \rho[QSQ] + \rho[SIQ] + \rho[SPQ] + g_P\theta[PS]\Theta([I]+[R]-I_{\mathrm{trig}}) \\
[\dot{QS_V}] &= g_E(1-\epsilon_2)[E_T S_V] - g_Q[QS_V] - \rho[QS_V] \\
&\quad - \tau_I[QS_V I] - \tau_P[QS_V P] - \rho[QS_V Q] + \rho[S_V IQ] \\
&\quad + \rho[S_V PQ] + g_P\theta[PS_V]\Theta([I]+[R]-I_{\mathrm{trig}}) - \frac{\nu}{[S_V]}[QS_V]\Theta([I]+[R]-I_{\mathrm{trig}})\Theta([S_V]) \\
[\dot{QV}] &= g_E(1-\epsilon_2)[E_T V] + g_E\epsilon_2[QE_T] + g_O\epsilon_1[QO] \\
&\quad - g_Q[QV] + \rho[VIQ] + \rho[VPQ] + g_P\theta[PV]\Theta([I]+[R]-I_{\mathrm{trig}}) \\
&\quad + \frac{\nu}{[S_V]}[QS_V]\Theta([I]+[R]-I_{\mathrm{trig}})\Theta([S_V]) \\
[\dot{RE}] &= g_I[IE] + g_Q[QE] - g_E[RE] - \rho[REQ] \\
&\quad + \tau_I[RSI] + \tau_P[RSP] + \tau_I[RS_V I] + \tau_P[RS_V P] \\
[\dot{RE_T}] &= g_I[IE_T] + g_Q[QE_T] - g_E(1-\epsilon_2)[RE_T] \\
&\quad - g_E\epsilon_2[RE_T] + \rho[REQ]
\end{aligned}
$$

$$
\begin{aligned}
[\dot{RI}] &= g_I[II] + g_Q[QI] - g_I[RI] + g_P(1-\theta)[RP] \\
&\quad - \rho[RIQ] \\
[\dot{RO}] &= g_I[IO] + g_Q[QO] - g_O(1-\epsilon_1)[RO] \\
&\quad - g_O\epsilon_1[RO] + \rho[RSQ] + \rho[RS_VQ] \\
[\dot{RP}] &= g_I[IP] + g_Q[QP] + g_E[RE] - g_P(1-\theta)[RP] \\
&\quad - \rho[RPQ] - g_P\theta[RP]\Theta([I]+[R]-I_{\mathrm{trig}}) \\
[\dot{RQ}] &= g_I[IQ] + g_Q[QQ] + g_E(1-\epsilon_2)[RE_T] \\
&\quad - g_Q[RQ] + \rho[RIQ] + \rho[RPQ] + g_P\theta[RP]\Theta([I]+[R]-I_{\mathrm{trig}}) \\
[\dot{RR}] &= g_I[IR] + g_Q[QR] + g_I[RI] + g_Q[RQ] \\
[\dot{RS}] &= g_I[IS] + g_Q[QS] + g_O(1-\epsilon_1)[RO] \\
&\quad - \tau_I[RSI] - \tau_P[RSP] - \rho[RSQ] \\
[\dot{RS}_V] &= g_I[IS_V] + g_Q[QS_V] - \tau_I[RS_VI] - \tau_P[RS_VP] \\
&\quad - \rho[RS_VQ] - \frac{\nu}{[S_V]}[RS_V]\Theta([I]+[R]-I_{\mathrm{trig}})\Theta([S_V]) \\
[\dot{RV}] &= g_I[IV] + g_Q[QV] + g_E\epsilon_2[RE_T] + g_O\epsilon_1[RO] \\
&\quad + \frac{\nu}{[S_V]}[RS_V]\Theta([I]+[R]-I_{\mathrm{trig}})\Theta([S_V]) \\
[\dot{SE}] &= g_O(1-\epsilon_1)[OE] - g_E[SE] - \tau_I[ESI] \\
&\quad - \tau_P[ESP] - \rho[ESQ] - \rho[SEQ] + \tau_I[SSI] \\
&\quad + \tau_P[SSP] + \tau_I[SS_VI] + \tau_P[SS_VP] \\
[\dot{SE}_T] &= g_O(1-\epsilon_1)[OE_T] - g_E(1-\epsilon_2)[SE_T] \\
&\quad - g_E\epsilon_2[SE_T] - \tau_I[E_TSI] - \tau_P[E_TSP] - \rho[E_TSQ] \\
&\quad + \rho[SEQ] \\
[\dot{SI}] &= g_O(1-\epsilon_1)[OI] - g_I[SI] - \tau_I[SI] \\
&\quad + g_P(1-\theta)[SP] - \tau_I[ISI] - \tau_P[ISP] \\
&\quad - \rho[ISQ] - \rho[SIQ]
\end{aligned}
$$

$$
\begin{aligned}
[\dot{SO}] &= g_O(1-\epsilon_1)[OO] - g_O(1-\epsilon_1)[SO] \\
&\quad - g_O\epsilon_1[SO] - \tau_I[OSI] - \tau_P[OSP] - \rho[OSQ] \\
&\quad + \rho[SSQ] + \rho[SS_VQ] \\
[\dot{SP}] &= g_O(1-\epsilon_1)[OP] + g_E[SE] - \tau_P[SP] \\
&\quad - g_P(1-\theta)[SP] - \tau_I[PSI] - \tau_P[PSP] \\
&\quad - \rho[PSQ] - \rho[SPQ] - g_P\theta[SP]\Theta([I]+[R]-I_{\text{trig}}) \\
[\dot{SQ}] &= g_O(1-\epsilon_1)[OQ] + g_E(1-\epsilon_2)[SE_T] \\
&\quad - g_Q[SQ] - \rho[SQ] - \tau_I[QSI] - \tau_P[QSP] \\
&\quad - \rho[QSQ] + \rho[SIQ] + \rho[SPQ] + g_P\theta[SP]\Theta([I]+[R]-I_{\text{trig}}) \\
[\dot{SR}] &= g_O(1-\epsilon_1)[OR] + g_I[SI] + g_Q[SQ] \\
&\quad - \tau_I[RSI] - \tau_P[RSP] - \rho[RSQ] \\
[\dot{SS}] &= g_O(1-\epsilon_1)[OS] + g_O(1-\epsilon_1)[SO] \\
&\quad - 2\tau_I[SSI] - 2\tau_P[SSP] - 2\rho[SSQ] \\
[S\dot{S}_V] &= g_O(1-\epsilon_1)[OS_V] - \tau_I[SS_VI] - \tau_P[SS_VP] \\
&\quad - \rho[SS_VQ] - \tau_I[S_VSI] - \tau_P[S_VSP] - \rho[S_VSQ] \\
&\quad - \frac{\nu}{[S_V]}[SS_V]\Theta([I]+[R]-I_{\text{trig}})\Theta([S_V]) \\
[\dot{SV}] &= g_O(1-\epsilon_1)[OV] + g_E\epsilon_2[SE_T] + g_O\epsilon_1[SO] \\
&\quad - \tau_I[VSI] - \tau_P[VSP] - \rho[VSQ] + \frac{\nu}{[S_V]}[SS_V]\Theta([I]+[R]-I_{\text{trig}})\Theta([S_V]) \\
[\dot{S_VE}] &= -g_E[S_VE] - \frac{\nu}{[S_V]}\Theta([I]+[R]-I_{\text{trig}})\Theta([S_V])[S_VE] \\
&\quad - \tau_I[ES_VI] - \tau_P[ES_VP] - \rho[ES_VQ] - \rho[S_VEQ] \\
&\quad + \tau_I[S_VSI] + \tau_P[S_VSP] + \tau_I[S_VS_VI] + \tau_P[S_VS_VP] \\
[\dot{S_VE_T}] &= -g_E(1-\epsilon_2)[S_VE_T] - g_E\epsilon_2[S_VE_T] \\
&\quad - \frac{\nu}{[S_V]}\Theta([I]+[R]-I_{\text{trig}})\Theta([S_V])[S_VE_T] - \tau_I[E_TS_VI] \\
&\quad - \tau_P[E_TS_VP] - \rho[E_TS_VQ] + \rho[S_VEQ]
\end{aligned}
$$

$$
\begin{aligned}
[S_V\dot{I}] =& -g_I[S_VI] - \tau_I[S_VI] - \frac{\nu}{[S_V]}\Theta([I]+[R]-I_{\mathrm{trig}})\Theta([S_V])[S_VI] \\
&+ g_P(1-\theta)[S_VP] - \tau_I[IS_VI] - \tau_P[IS_VP] \\
&- \rho[IS_VQ] - \rho[S_VIQ] \\
[S_V\dot{O}] =& -g_O(1-\epsilon_1)[S_VO] - g_O\epsilon_1[S_VO] \\
&- \frac{\nu}{[S_V]}\Theta([I]+[R]-I_{\mathrm{trig}})\Theta([S_V])[S_VO] - \tau_I[OS_VI] \\
&- \tau_P[OS_VP] - \rho[OS_VQ] + \rho[S_VSQ] + \rho[S_VS_VQ] \\
[S_V\dot{P}] =& g_E[S_VE] - \tau_P[S_VP] - g_P(1-\theta)[S_VP] \\
&- \tau_I[PS_VI] - \tau_P[PS_VP] - \rho[PS_VQ] - \rho[S_VPQ] \\
&- g_P\theta[S_VP]\Theta([I]+[R]-I_{\mathrm{trig}}) - \frac{\nu}{[S_V]}[S_VP]\Theta([I]+[R]-I_{\mathrm{trig}})\Theta([S_V]) \\
[S_V\dot{Q}] =& g_E(1-\epsilon_2)[S_VE_T] - g_Q[S_VQ] - \rho[S_VQ] \\
&- \tau_I[QS_VI] - \tau_P[QS_VP] - \rho[QS_VQ] + \rho[S_VIQ] \\
&+ \rho[S_VPQ] + g_P\theta[S_VP]\Theta([I]+[R]-I_{\mathrm{trig}}) - \frac{\nu}{[S_V]}[S_VQ]\Theta([I]+[R]-I_{\mathrm{trig}})\Theta([S_V]) \\
[S_V\dot{R}] =& g_I[S_VI] + g_Q[S_VQ] - \tau_I[RS_VI] - \tau_P[RS_VP] \\
&- \rho[RS_VQ] - \frac{\nu}{[S_V]}[S_VR]\Theta([I]+[R]-I_{\mathrm{trig}})\Theta([S_V]) \\
[S_V\dot{S}] =& g_O(1-\epsilon_1)[S_VO] - \tau_I[SS_VI] - \tau_P[SS_VP] \\
&- \rho[SS_VQ] - \tau_I[S_VSI] - \tau_P[S_VSP] - \rho[S_VSQ] \\
&- \frac{\nu}{[S_V]}[S_VS]\Theta([I]+[R]-I_{\mathrm{trig}})\Theta([S_V]) \\
[S_V\dot{S}_V] =& -2\tau_I[S_VS_VI] - 2\tau_P[S_VS_VP] - 2\rho[S_VS_VQ] \\
&- \frac{2\nu[S_VS_V]\Theta([I]+[R]-I_{\mathrm{trig}})\Theta([S_V])}{[S_V]} \\
[S_V\dot{V}] =& g_E\epsilon_2[S_VE_T] + g_O\epsilon_1[S_VO] - \tau_I[VS_VI] - \tau_P[VS_VP] \\
&- \rho[VS_VQ] + \frac{\nu}{[S_V]}[S_VS_V]\Theta([I]+[R]-I_{\mathrm{trig}})\Theta([S_V]) \\
&- \frac{\nu}{[S_V]}[S_VV]\Theta([I]+[R]-I_{\mathrm{trig}})\Theta([S_V])
\end{aligned}
$$

$$
\begin{aligned}
[\dot{VE}] &= g_E\epsilon_2[E_TE] + g_O\epsilon_1[OE] - g_E[VE] - \rho[VEQ] \\
&+ \tau_I[VSI] + \tau_P[VSP] + \tau_I[VS_VI] + \tau_P[VS_VP] \\
&+ \frac{\nu}{[S_V]}[S_VE]\Theta([I]+[R]-I_{\text{trig}})\Theta([S_V]) \\
[\dot{VE_T}] &= g_E\epsilon_2[E_TE_T] + g_O\epsilon_1[OE_T] - g_E(1-\epsilon_2)[VE_T] \\
&- g_E\epsilon_2[VE_T] + \rho[VEQ] + \frac{\nu}{[S_V]}[S_VE_T]\Theta([I]+[R]-I_{\text{trig}})\Theta([S_V]) \\
[\dot{VI}] &= g_E\epsilon_2[E_TI] + g_O\epsilon_1[OI] - g_I[VI] + g_P(1-\theta)[VP] \\
&- \rho[VIQ] + \frac{\nu}{[S_V]}[S_VI]\Theta([I]+[R]-I_{\text{trig}})\Theta([S_V]) \\
[\dot{VO}] &= g_E\epsilon_2[E_TO] + g_O\epsilon_1[OO] - g_O(1-\epsilon_1)[VO] \\
&- g_O\epsilon_1[VO] + \rho[VSQ] + \rho[VS_VQ] + \frac{\nu}{[S_V]}[S_VO]\Theta([I]+[R]-I_{\text{trig}})\Theta([S_V]) \\
[\dot{VP}] &= g_E\epsilon_2[E_TP] + g_O\epsilon_1[OP] + g_E[VE] - g_P(1-\theta)[VP] \\
&- \rho[VPQ] - g_P\theta[VP]\Theta([I]+[R]-I_{\text{trig}}) + \frac{\nu}{[S_V]}[S_VP]\Theta([I]+[R]-I_{\text{trig}})\Theta([S_V]) \\
[\dot{VQ}] &= g_E\epsilon_2[E_TQ] + g_O\epsilon_1[OQ] + g_E(1-\epsilon_2)[VE_T] \\
&- g_Q[VQ] + \rho[VIQ] + \rho[VPQ] + g_P\theta[VP]\Theta([I]+[R]-I_{\text{trig}}) \\
&+ \frac{\nu}{[S_V]}[S_VQ]\Theta([I]+[R]-I_{\text{trig}})\Theta([S_V]) \\
[\dot{VR}] &= g_E\epsilon_2[E_TR] + g_O\epsilon_1[OR] + g_I[VI] + g_Q[VQ] \\
&+ \frac{\nu}{[S_V]}[S_VR]\Theta([I]+[R]-I_{\text{trig}})\Theta([S_V]) \\
[\dot{VS}] &= g_E\epsilon_2[E_TS] + g_O\epsilon_1[OS] + g_O(1-\epsilon_1)[VO] \\
&- \tau_I[VSI] - \tau_P[VSP] - \rho[VSQ] + \frac{\nu}{[S_V]}[S_VS]\Theta([I]+[R]-I_{\text{trig}})\Theta([S_V]) \\
[\dot{VS_V}] &= g_E\epsilon_2[E_TS_V] + g_O\epsilon_1[OS_V] - \tau_I[VS_VI] - \tau_P[VS_VP] \\
&- \rho[VS_VQ] + \frac{\nu}{[S_V]}[S_VS_V]\Theta([I]+[R]-I_{\text{trig}})\Theta([S_V]) \\
&- \frac{\nu}{[S_V]}[VS_V]\Theta([I]+[R]-I_{\text{trig}})\Theta([S_V]) \\
[\dot{VV}] &= g_E\epsilon_2[E_TV] + g_O\epsilon_1[OV] + g_E\epsilon_2[VE_T] + g_O\epsilon_1[VO] \\
&+ \frac{\nu}{[S_V]}[S_VV]\Theta([I]+[R]-I_{\text{trig}})\Theta([S_V]) + \frac{\nu}{[S_V]}[VS_V]\Theta([I]+[R]-I_{\text{trig}})\Theta([S_V])
\end{aligned}
$$
